# Physiological Role of Kv1.3 Channel in T Lymphocyte Cell Investigated Quantitatively by Kinetic Modeling

**DOI:** 10.1371/journal.pone.0089975

**Published:** 2014-03-03

**Authors:** Panpan Hou, Rong Zhang, Yongfeng Liu, Jing Feng, Wei Wang, Yingliang Wu, Jiuping Ding

**Affiliations:** 1 Key Laboratory of Molecular Biophysics of the Ministry of Education, College of Life Science and Technology, Huazhong University of Science and Technology, Wuhan, Hubei, China; 2 State Key Laboratory of Virology, College of Life Sciences, Wuhan University, Wuhan, China; 3 School of Public Health, Xinxiang Medical University, Xinxiang, Henan, China; University of Houston, United States of America

## Abstract

Kv1.3 channel is a delayed rectifier channel abundant in human T lymphocytes. Chronic inflammatory and autoimmune disorders lead to the over-expression of Kv1.3 in T cells. To quantitatively study the regulatory mechanism and physiological function of Kv1.3 in T cells, it is necessary to have a precise kinetic model of Kv1.3. In this study, we firstly established a kinetic model capable to precisely replicate all the kinetic features for Kv1.3 channels, and then constructed a T-cell model composed of ion channels including Ca^2+^-release activated calcium (CRAC) channel, intermediate K^+^ (IK) channel, TASK channel and Kv1.3 channel for quantitatively simulating the changes in membrane potentials and local Ca^2+^ signaling messengers during activation of T cells. Based on the experimental data from current-clamp recordings, we successfully demonstrated that Kv1.3 dominated the membrane potential of T cells to manipulate the Ca^2+^ influx via CRAC channel. Our results revealed that the deficient expression of Kv1.3 channel would cause the less Ca^2+^ signal, leading to the less efficiency in secretion. This was the first successful attempt to simulate membrane potential in non-excitable cells, which laid a solid basis for quantitatively studying the regulatory mechanism and physiological role of channels in non-excitable cells.

## Introduction

Kv1.3 and IK (K_Ca_3.1) are two kinds of potassium channels in T cells. Kv1.3 channels are activated upon the depolarization of the membrane potential, while IK channels are activated by the Calcium ion [1]–[3]. There are three types of T cells, Naïve T cell, T_CM_ and T_EM_ cells [3]. These quiescent cells exhibit a similar K^+^ channel expression pattern with ∼300 Kv1.3 and ∼10 IK channels per cell. However, Kv1.3 channels are up regulated to ∼1500 in the activated T_EM_ effectors and IK channels are up regulated to ∼500 in the activated T_CM_ effectors [3]. Such high expression of Kv1.3 channels has been reported associated with many chronic inflammatory and autoimmune disorders such as multiple sclerosis (MS), type 1 diabetes mellitus (T1DM) and rheumatoid arthritis (RA) [4]–[7], and therefore Kv1.3 channel served as a potential therapeutic target for treatment of these diseases, which was indicated by the blockers of chemical molecules and peptide toxins [8]–[11].

Kv1.3 channel is a voltage-activated K^+^ channel that shows a fast activation and slow C-type inactivation and recovery [2], . Comparing with other ion channels, Kv1.3 is sensitive to many pharmacological agents including small organic compounds and many peptide toxins such as margatoxin (MgTx), agitoxin-2 (AgTx2), ShK etc. [15], [16]. Particularly, one engineered scorpion toxin ADWX-1 (autoimmune drug from Wenxin group) shows the highest affinity to Kv1.3 channels in picomolar range of potency [17]. Those properties enable us to identify the Kv1.3 from various lymphocyte K^+^ currents.

Besides Kv1.3, five main types of ion channels have been identified at the molecular level in T cells. They are Ca^2+^-release activated calcium (CRAC) channel, intermediate K^+^(IK) channel, TASK channel (a two-pore domain potassium (K_2P_) channel), TRPM7 channel and Osmo-activated Cl^−^ (Cl_swell_) channel [3]. As a major calcium source in T lymphocyte cells, the CRAC channel, formed by the STIM1 and Orai1 subunits, leads a Ca^2+^ influx while depleting the endoplasmic reticulum (ER) Ca^2+^ store [18]–[20]. Since the intracellular Ca^2+^ can modulate various important physiological functions such as potassium channel gene expression and secretion, the Ca^2+^ influx via CRAC channels may form a positive feedback to induce the larger Ca^2+^ signal [3], [18], [21].

It is well-known that a typical resting effector memory T (T_EM_) cell contains ∼300 functional Kv1.3 channels and ∼10 functional IK channels on the surface membrane [2], [3], which can prevent membrane potential from excessively larger depolarization [18], [19], [22]–[25]. During T cell activation, they are the major contributor to maintain the membrane potential that promotes Ca^2+^ influx. A negative membrane potential enhances Ca^2+^ entry by optimizing the electrochemical driving force for Ca^2+^ movement through CRAC channels [26]–[28], and inhibit the Kv1.3 and IK potassium channels by their specific inhibitors will significantly reduce the calcium signaling [8], [9]. However, a clear pattern in quantitative description on the functional role that Kv1.3 channel plays in regulating the membrane potential and the intracellular local and global Ca^2+^ signaling remains wrapped.

Kinetic modeling provides a good way for studying and predicting the kinetic behavior of ion channels and their function in cells. Several kinetic models of Kv1.3 channel have been reported previously [12]–[14]. They did excellent works focused on the individual activation, inactivation or recovery characteristics. The accuracy of the cell model is based on the comprehensiveness of the ion channel models it constitutes. In this study, we establish a novel Kv1.3 model capable to precisely describe the whole kinetic behavior of Kv1.3, using a software CeL [29]. Based on the Hodgkin-Huxley theory, a model cell with appropriate component of channels can be used to simulate the firing pattern of action potentials in excitable cells [30]. But the H-H model has never been used to simulate the membrane potentials in non-excitable cells. This is a first attempt to construct a T-cell model, composed of several model channels including Kv1.3, CRAC, IK and TASK channels, for mimicking the dynamic behavior of membrane potentials and intracellular Ca^2+^ signaling in T cells. Although there is no action potential in T lymphocyte cells, it is still interesting to know the membrane potential performances after stimulation. Combined with the current-clamp experimental data with different amount of Kv1.3 channels blocked by ADWX-1 from T lymphocyte cells, we do quantitatively mimic all the changes in membrane potential and the corresponding Ca^2+^ signal in the non-excitable model T-cell. Overall, a simulation framework has been provided for further studying the regulatory mechanism of other channels in T lymphocyte cells, which lays a solid basis for both the immunological and ion-channel fields.

## Materials and Methods

### Ethics Statement

Peripheral venous blood was obtained from healthy volunteers, who provided their written informed consent to participate in this study. The consent procedure and our research were approved by the Ethics Committee of the College of Life Sciences in Wuhan University (Permit Number: ECCLS 20120076).

### Cell culture and Transfection

Full length cDNA for Kv1.3 was subcloned into pcDNA3.1/Zeo (Invitrogen). HEK293 cells were cultured in modified Eagle's medium (DMEM, Gibco) supplemented with 10% fetal bovine serum (FBS, Gibco) at 37°C incubator with 5% CO_2_. The day before transfection, cells were transfered into a 24-well plate and transiently transfected using lipofectamine 2000 (Invitrogen) according to manufacturer's protocol. Recordings were carried out in 1–2 days after transfection.

### Preparation of human T lymphocyte cells

Peripheral venous blood was obtained from healthy volunteers. Mononuclear cells were isolated by Ficoll-Hypaque density gradient. T cells were separated from PBMCs by using FACS with PE conjugated CD3^+^ antibody and maintained in RPMI 1640 medium supplemented with 1 mM L-glutamine and 10% fetal bovine serum (Gibco) in a humidified, 5% CO2 incubator at 37°C. Recordings were carried out in 24 hours after T lymphocyte cells preparation.

### Solutions

For whole-cell patch recordings, the pipette solution contained the following (in mM): 130 KCl, 2.5 MgCl_2_, 10 HEPES, 1 EGTA, 2 K_2_ATP (pH 7.4) titrated with KOH, bath solution contained the following (in mM): 145 NaCl, 5 KCl, 1 MgCl_2_, 2 CaCl_2_, 10 HEPES (pH 7.4) titrated with NaOH. All the chemicals were attained from Sigma.

### Electrophysiology

Patch pipettes pulled from borosilicate glass capillaries with resistance of 2–3megohms in transfected HEK293 cell experiments and 6–8megohmsin T lymphocyte cell experiments when filled with pipette solution. All experiments were performed using an EPC-9 patch-clamp amplifier and corresponding software (HEKA, Germany). Currents were typically digitized at 20 kHz and filtered at 2.9 kHz (Bessel). The 85% of series resistances were electronically compensated. During recording, the corresponding solution was puffed onto cells via a puffer pipette containing seven solution channels. All experiments were performed at room temperature (20–24°C).

### Intracellular Ca^2+^ Measurements

T lymphocyte cells were incubated in 100 µl of extracellular solution (ECS) containing 140 mM NaCl, 5 mM KCl, 1 mM MgCl_2_, 1.8 mM CaCl_2_, 10 mM Hepes (pH 7.4), 10 mM glucose, 2 µM fura-2/AM (Invitrogen), 2 mM Probenecid, 0.05% Pluronic F-127 and 0.1% bovine serum albumin at 37°C for 30 min. After the incubation, cells were placed in the platform for optical imaging. The imaging of intracellular Ca^2+^ was performed on Olympus-IX70 microscope system with a polychromatic light source (T.I.L.L. Photonics GmbH, Grafelfing, Germany). The excitation wavelengths of fura-2 fluorescence were 340 nm and 380 nm, coming from the bottom of plate at 1 Hz. All of the experiments were performed at room temperature.

### Data analysis

Recording data were analyzed with IGOR (Wavemetrics, Lake Oswego, OR), Clampfit (Molecular Devices, Inc.) and Sigmaplot software (SPSS, Inc.). Unless stated otherwise, the data are presented as mean ± S.D. The conductance of Kv1.3 channels was calculated from G = *I_peak_/(V-E_kv1.3_)*, where *I_peak_* is the peak current, *E_kv1.3_* is the reversal potential of Kv1.3. The conductance-voltage (G-V) curves of activation were fitted by the Boltzmann equation:
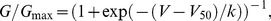
(1)Where *G_max_* is the maximum conductance, *V_50_* is the half maximal conductance voltage and *κ* is a slope factor. The steady-state inactivation curve was fitted to the Boltzmann equation:
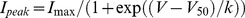
(2)Where *I_max_* is the maximal peak current, *V_50_* is the half maximal availability voltage and the *κ* is a slope factor.

### Mathematical modeling and simulation

T lymphocyte model cell has been described as the following equations [31]:
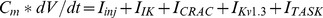
(3)where *V* is the voltage in mV, *C_m_* is the capacity of the cell membrane in pF, *t* is the time in ms, *I_inj_* is the injection current in pA, *I_TASK_* is the leak current in pA, *I_CRAC_* is the CRAC current in pA, *I_Kv1.3_* is the Kv1.3 current in pA and *I_IK_* is the IK current in pA. In this study, *C_m_* = 1.5 pF and the resting potential *V_Rest_ = −55 mV* [32], [33].

All currents except *I_inj_* can be written as follows:
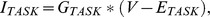
Where the total conductance *G_TASK_* = 0.13 nS [34] and the reversal potential *E_TASK_* = −100 mV [35];
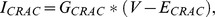
Where the total conductance *G_CRAC_* = 0.09 nS [3] and the reversal potential *E_CRAC_* = +80 mV [3];
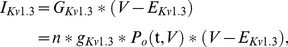
(4)Where the total conductance *G_Kv1.3_* = Variable, *n* is the number of the *K_v1.3_* channel, the a single-channel conductance *g_Kv1.3_* = 15 pS [36], and the reversal potential *E_Kv1.3_* = −73 mV [37], and *P_o_*(*V*) comes from the *K_v1.3_* model.
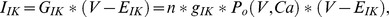
where the single-channel conductance *g_IK_* = 11 pS in normal Ringer saline [38], *n* is the number of the *IK* channel, the total conductance *G_IK_* = 0.11 nS, assuming that 10 channels per T_EM_ cell [3] and the reversal potential *E_IK_* = −75 mV [31], and *P_o_*(*Ca*) comes from the *P_o_*(*Ca*)-[*Ca^2+^*]*_i_* curve reported by Grissmer et al. [38].

Considering that the *IK* channel is triggered by a global Ca^2+^ concentration in nM, which usually is tremendously smaller than the local Ca^2+^ in µM [39], we thus determined the global Ca^2+^ concentration based on an assumption proportional to the local Ca^2+^ with a average intracellular Ca^2+^ (about 500 nM) after CRAC channel opening, which could be measured in T cells [40].

To trigger the secretion in T cells, we need to calculate the local Ca^2+^ concentration, depending strictly on net influx and local diffusion of Ca^2+^ near the CRAC channel in T cells. Assuming that secretory proteins, i.e. complexes of synaptobrevin, syntaxin and SNAP-25 termed SNARE complexes are colocalized to individual Ca^2+^ sources such as CRAC channel in T cells [41], [42], we employ a formulation first utilized by Beeler and Reuter [43] to convert Ca^2+^ influx to a local Ca^2+^ concentration:
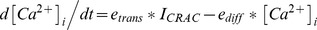
(5)where [*Ca^2+^*]*_i_* is the intracellular Ca^2+^ concentration, e_trans_ is an arbitrary transfer coefficient that scales calcium influx to the calcium concentration, and e_diff_ is the calcium diffusion coefficient [44]. In this study, e_trans_ = 0.006 µM*pA^−1^*ms^−1^ and e_diff_ = 0.003 ms^−1^ [29], which should be able to produce a local Ca^2+^ concentration high enough (≥10 µM) to trigger secretion [45]. Therefore, we can avoid introducing more hypotheses like many uncertain diffusion factors, unknown dissociation equilibrium constant K_d_ and quantity of various buffers and the calcium efflux releasing from stores. All of those factors can be simply solved by changing the diffusion and transfer coefficients to reveal Ca^2+^ properties in T cells.

The differential equations for the kinetic modeling were solved numerically, using a Q-Matrix or Five-order Runge-Kutta integration method. The fitting procedure is based on a PSO-GSS algorithm for direct estimation of rate constants from macroscopic currents [29]. The integrating routines were written and executed with software CeL (HUST, Wuhan, Hubei, China), compiled with the C++ compiler to run under Windows XP [31]. Kinetic parameters were optimized with CeL as previously described [29].

## Results

To understand the role Kv1.3 channels play in T cells, it is extremely necessary to establish a kinetic model capable to precisely describe its detailed kinetic characteristics under physiological conditions. After that, we can use this Kv1.3 channel model and other four channel models to construct a model T-cell for mimicking the behaviors of membrane potentials and the intracellular Ca^2+^ signaling and thereby to determine Kv1.3 channel physiological role in T cells.

### A sequential kinetic model of Kv1.3 channels

**Figure 1:**
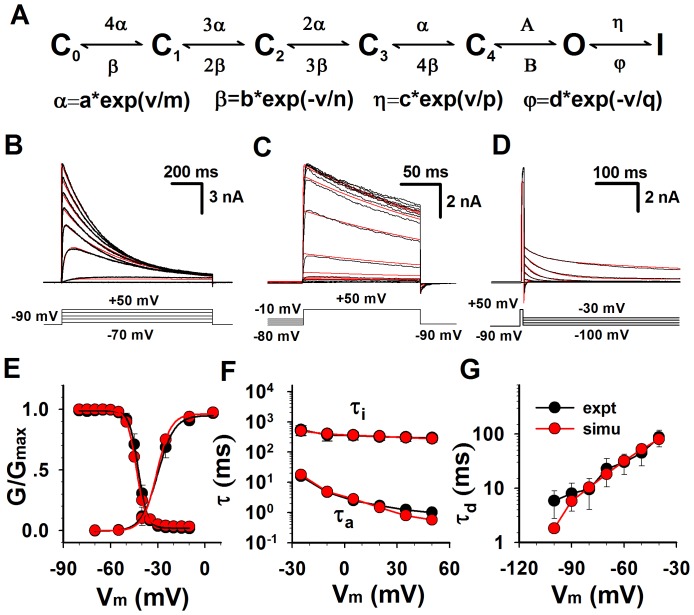
Comparison of the kinetic characteristics of Kv1.3 channels between the experimental data and simulations. (**A**) Kinetic model for Kv1.3 channels. The voltage-dependent rates α, β,η and φ are in an exponential form, in which the a, b, c and d are the pre-exponential factor in ms^−1^ and the m, n, p and q are the exponential factor in mV; the rates A and B are constants in ms^−1^; the v is voltage in mV. (**B**) The activation currents of Kv1.3 channels expressed in HEK 293 cells were recorded from a whole-cell patch. Activation was elicited by voltage steps from −70 to +50 mV with increment of 15 mV from holding potential at −90 mV applied every 90 s. The voltage protocol is plotted at the bottom. Red lines are model simulations (N = 7465). (**C**) Steady-state inactivation was determined by measuring the peak current at +50 mV, following the 60-s pre-conditional pulses between −80 mV and −10 mV in increments of 5 mV. The voltage protocol is plotted at the bottom. Red lines are model simulations (N = 4518). (**D**) Cells were depolarized to +50 mV for 7 ms from a holding −90 mV and then repolarized to various voltages between −100 and −30 mV in increments of 10 mV. The voltage protocol is placed at the bottom. Red lines are model simulations (N = 4605). (**E**) Statistics of activation and steady-state inactivation. For activation, V_50_ = −28.4±1.6 mV, s = 4.9±0.5 mV (n = 9); for inactivation, V_50_ = −43.5± 3.7 mV, s = 3.0±0.3 mV (n = 5). (**F**) Time constants of activation (n = 9) and inactivation (n = 5) were plotted as the function of voltages. (**G**) Time constants of deactivation (n = 10) were plotted as the function of voltages. The red and black lines (or symbols) are for the simulations and experimental data, respectively.

Several non-sequential kinetic models of Kv1.3 had been reported for simulating the kinetics of Kv1.3 currents, but they focused on the individual activation, inactivation or recovery characteristics [2], [12]–[14]. Here we provided a simple model for simultaneously simulating the whole kinetics of Kv1.3 currents including activation, deactivation, steady-state inactivation and recovery (Fig. 1A). It is a sequential model of which the activation process depends on the forward (activation) rates α and A from the closed (C_i_) to the open (O) to the inactivated (I) states, the deactivation process depends on the backward rates β and B from O to C_i_ and the inactivation process depends on the rates η and φ from O to I. All the rates can be determined by automatically fitting the model to the experimental data with the software CeL [29].

Many studies have confirmed that the heterologously expressed Kv1.3 channels possess biophysical and pharmacological signature closely resemble that of native Kv1.3 channels in T lymphocytes cells [37], [46], [47]. Their voltage dependence, rates of opening and closing, single-channel conductance, inactivation properties, and blockade by different compounds including tetraethylammonium (TEA) and charybdotoxin (CTX) were indistinguishable from native Kv1.3 channels. So we recorded Kv1.3 dynamics from transfected HEK293 cells to simplify the modeling.

To complete the kinetic model of Kv1.3, experiments were performed on Kv1.3-transfected HEK293 cells to acquire the activation (Fig. 1B), steady-state inactivation (Fig. 1C) and deactivation (Fig. 1D), respectively. In Fig. 1B, Kv1.3 currents (black) exhibited a fast activation and then a slow inactivation, evoked by 1 s depolarizing voltage steps ranging from −70 to +50 mV in 15 mV increments from a 90 s holding potential of −90 mV to remove the possible inactivation. The forward rates of the process C→O→I could be determined by fitting it to the activation currents. Here the red lines conferred a perfect fit (Fig. 1B). The voltage dependence of steady-state inactivation currents (black), standing for the availability of channels, was obtained by applying a set of conditioning voltages ranging from −80 to −10 mV for 90 s in 5 mV increments and then measured at a 150 ms test pulse of 50 mV as indicated (Fig. 1C). The backward rates of C←O←I could be determined by fitting it to the steady-state inactivation currents. In Fig. 1C, all the fits (red) were close to the data (black). For deactivation currents (black), cells were depolarized to +50 mV for 7 ms from a holding potential of −90 mV and then repolarized to various voltages between −100 and −30 mV in 10 mV increments (Fig. 1D). Similarly, the backward rate of C←O could be determined by fitting it to deactivation currents. All the fits (red) were fully overlapping to the data (black) (Fig. 1D).

The normalized activation (G-V) curves (black) of Kv1.3 had an averaged value of V_50_ = −28.4±1.6 mV, and the normalized steady-state inactivation (availability) curve tested at 50 mV had a V_50_ = −43.5±3.7 mV (Fig. 1E left), consistent with previous work on Kv1.3 [37], [46], [47]. In Fig. 1E, both the G-V and steady-state curves from simulations (red) were closely coincides with that of data (black). Additionally, the time constants of activation (τ_a_) (Fig. 1F bottom), inactivation (τ_i_) (Fig. 1F top), and deactivation (τ_d_) (Fig. 1G) were also consistent to the experimental data.

**Figure 2:**
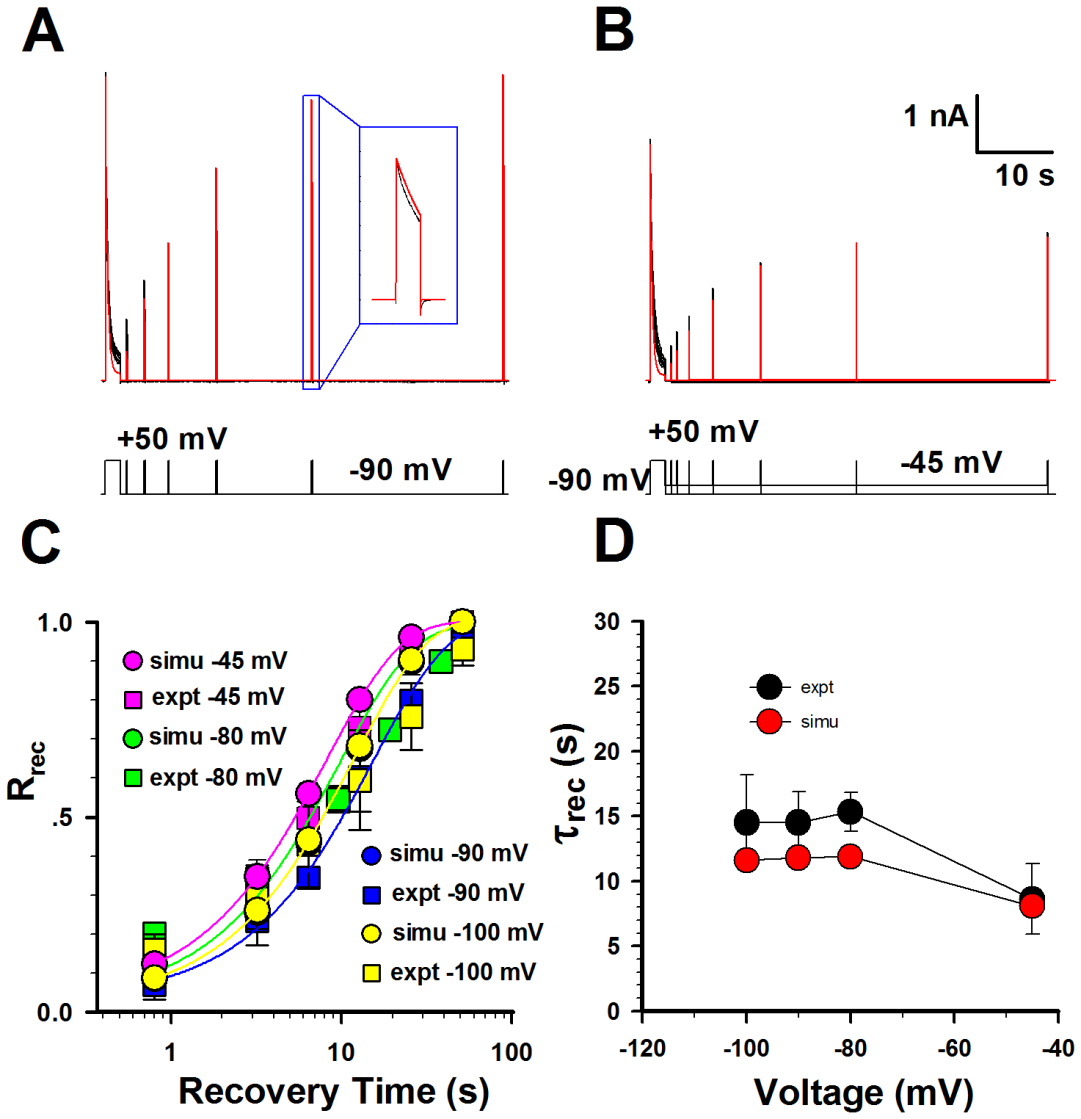
Comparison of the recovery of Kv1.3 channels between the experimental data and simulations. (**A–B**) The representative currents of recovery from inactivation were obtained in whole-cell patch mode at −90 mV and −45 mV. Two-pulse protocol: from a holding potential of −90 mV, the first pulse 1 (P1) was applied for 1 s at +50 mV, and then the second +50-mV pulse (P2) was applied for 150 ms after a variable interpulse interval at −90 mV in (**A**) and −45 mV in (**B**). The detailed recovery currents are shown in the box. The voltage protocol is placed at the bottom. Red lines are fits and black ones are data. The channel numbers for simulations are 3788 for −90 mV and 2740 for −45 mV. (**C**) Comparison of the time courses of recovery between the experimental data (black) and the simulations (red) at −100 mV, −90 mV, −80 mV and −45 mV. (**D**) Comparison of time constants of recovery between the experimental data (black) and the simulations (red). The time constants of recovery (mean ± SEM) are 8.6±2.7 s (n = 5) at −45 mV, 15.6±1.6 s (n = 3) at −80 mV, 14.6±2.4 s (n = 7) at −90 mV, 14.6±4.7 s (n = 3) at −100 mV, respectively.

Based on the perfect fitness to the activation, inactivation, deactivation and steady-state inactivation data, all the parameters in Kv1.3 model were basically determined. Then we further examined whether this model matched the data of recovery from inactivation by subtly readjusting the parameters in the Kv1.3 model. Since the time course of recovery from inactivation is mono-exponential, the sequential model with only one inactivation state is good enough for the Kv1.3 kinetics. This model conferred by and large a rough fit to both the currents recovering at −45 and −90 mV. Fig. 2C displayed a set of fractional recovery curves arising from a two-pulse protocol (P1 = 1 s, P2 = 150 ms, V = −100, −90, −80 and −45 mV). The time constants of recovery were 8.6 s at −45 mV, 15.6 s at −80 mV, 14.6 s at −90 mV, 14.6 s at −100 mV, approximately consistent to the experimental data (Fig. 2D). Interestingly, this result indicated that the recovery of Kv1.3 was weakly voltage-dependent under physiological condition [14]. So far, a sequential kinetic model, well illuminating the whole kinetic behavior of Kv1.3 channels, was successfully established. All the parameters in model were listed in Table 1.

**Table 1:** Parameters of Kv1.3 model.

Parameter	Values
a	0.448(ms^−1^)
b	0.043(ms^−1^)
c	0.003(ms^−1^)
d	0.00008(ms^−1^)
A	280.035(ms^−1^)
B	1.648(ms^−1^)
m	27.530(mV)
n	17.528(mV)
p	174.961(mV)
q	1016.330(mV)

### Scorpion toxin ADWX-1 is a pore blocker of Kv1.3 channel without affecting its kinetics

**Figure 3:**
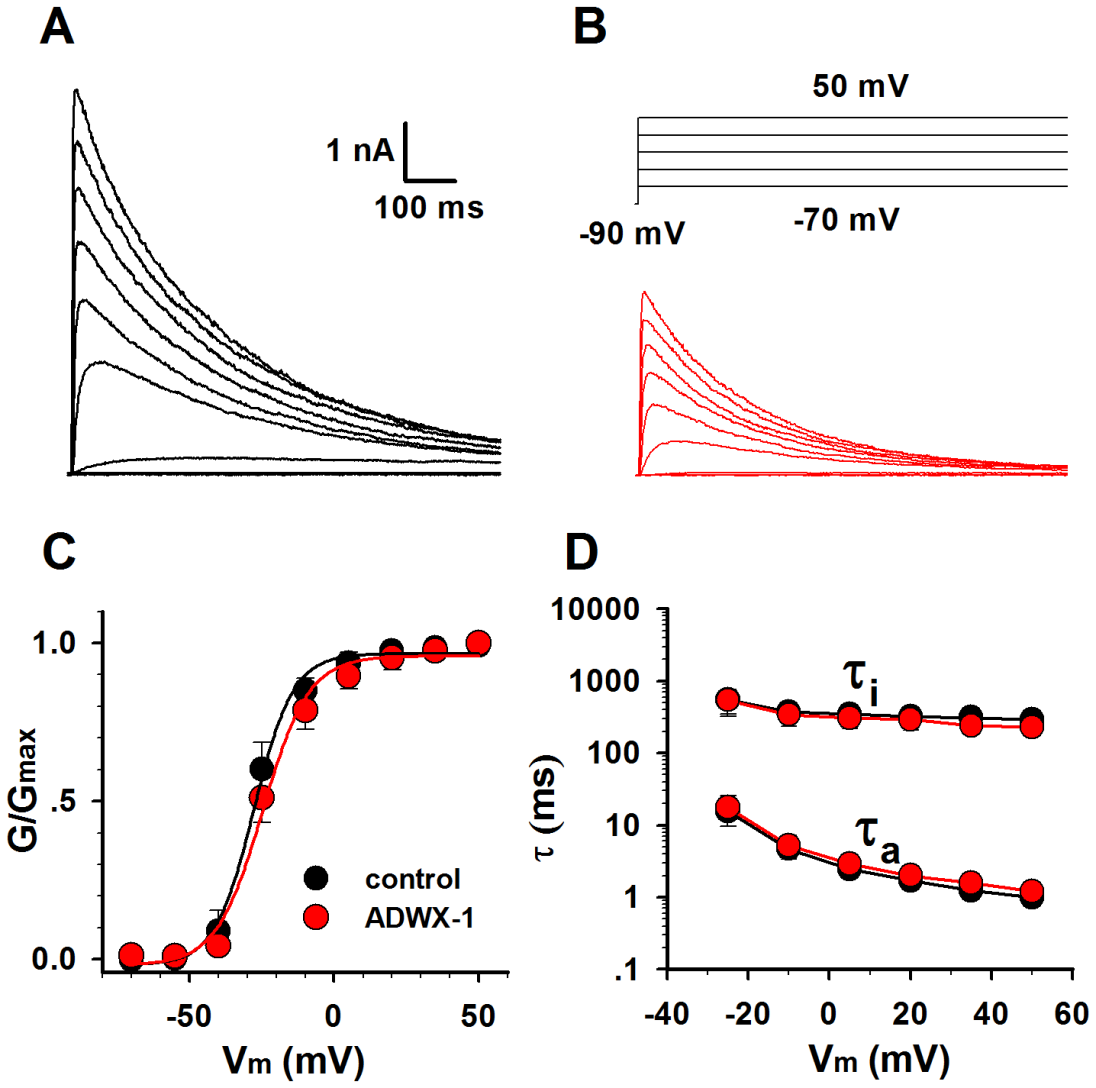
The ADWX-1 toxin blocked the Kv1.3 currents without changing the channel kinetics. (**A–B**) Kv1.3 activation currents were recorded before (black) and after (red) applying with 30 pM ADWX-1. Voltage protocol is placed at the top. (**C**) The activation G-V curves of Kv1.3 (n = 6), before (black) and after (red) applying with 30 pM ADWX-1. (**D**) The time constants of activation and inactivation of Kv1.3 (n = 6), before (black) and after (red) applying with 30 pM ADWX-1.

As we reported previously, the engineered scorpion toxin ADWX-1 is a potent specific inhibitor of Kv1.3. It has the highest affinity in ∼pM to Kv1.3 channels [17]. In Fig. 3A–B, 30 pM ADWX-1 blocked the Kv1.3 currents without changing the G-V curve (Fig. 3C) and the time constants of activation and inactivation (Fig. 3D). This means that the inhibition by ADWX-1 only reduces the number of open channels without affecting the channel kinetics. In other words, we can explore the Kv1.3 role in T cell by simply changing the number of Kv1.3 channels in applying with different ADWX-1 concentrations.

### Inhibition and characterization of Kv1.3 channels in T cells

**Figure 4:**
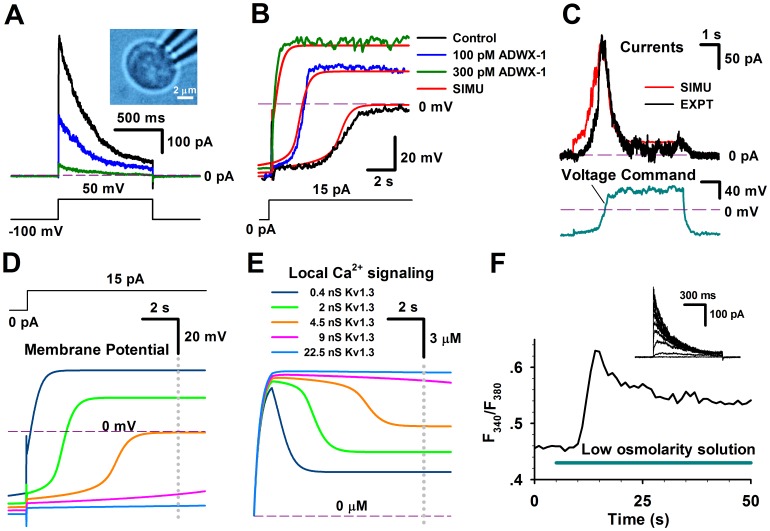
The number of Kv1.3 channels regulates the membrane potential of native T lymphocyte cells. (**A**) The Kv1.3 currents in a T lymphocyte cell, bathed in normal saline, were stimulated by a voltage step from −100 to 50 mV in the whole-cell mode, in the presence of 0 (black), 30 (blue) and 300 (green) pM ADWX-1. The inset shows a picture of a T cell. The measured membrane capacitance *C_m_* = 1.52 pF. The voltage protocol is placed at the bottom. (**B**) A T lymphocyte cell showed the changes in membrane potentials by an injection current of 15 pA, in the presence of 0 (black), 30 (blue) and 300 (green) pM ADWX-1, respectively. A model T cell, composed of Kv1.3, CRAC, IK and TASK channels, conferred the simulations (red) corresponding to the changes of membrane potentials resulted from the Kv1.3 conductance of 0.4, 2.0 and 4.5 nS, respectively. The resting potential of model cells was −55 mV. (**C**) The Kv1.3 currents from a native T lymphocytes cell (black) and a model T lymphocytes cell (red) were respectively elicited by a voltage command (cyan), conferring a number of Kv1.3 n = 330. Here the background currents were subtracted by 1 nM ADWX-1. (**D**) Simulation for membrane potentials in a model T cell stimulated by an injection current of 15 pA, while varying the number of Kv1.3 channels. The steady-state values (V_ss_) of membrane potentials went up to 35.7 mV for 0.4 nS (dark blue), 19.8 mV for 2 nS (green), −0.62 mV for 4.5 nS (dark yellow), −35.9 mV for 9 nS (pink) and −43.1 mV for 22.5 nS (light blue) at the time of 10 s (gray dotted line), respectively. (**E**) Simulations of intracellular calcium concentrations with the different numbers of Kv1.3 channels. The steady-state calcium concentrations [Ca^2+^]_ss_ were 3.48 µM for 0.4 nS (dark blue), 4.89 µM for 2 nS (green), 7.01 µM for 4.5 nS (dark yellow), 10.47 µM for 9 nS (pink) and 11.48 µM for 22.5 nS (light blue) at the moment of 10 s (gray dotted line), respectively. (**F**) The time course of the intracellular Ca^2+^ concentrations, stimulated by low osmolarity solution labeled in a cyan bar, in a native T lymphocyte cell. The inset shows the Kv1.3 currents recorded from the same T lymphocyte cell.

Based on the sequential kinetic model of Kv1.3 channels, their inhibition features were further investigated in T cells. We first examined the voltage-activated currents in T cells in the absence and presence of 30 and 300 pM ADWX-1. Our results demonstrated that the slow inactivated currents were sensitive to ADWX-1 (Fig. 4A). And after applying 300 pM ADWX-1 to T cell, there is little outward current left at 50 mV, indicating that Kv1.3 current was the major prominent of outward current in this case. In current-clamp experiments, we also found that the membrane potentials of T cells rapidly went to depolarization direction by 15 pA current injection while Kv1.3 channel were inhibited by ADWX-1 (Fig. 4B, green and blue), in contrast, the membrane potential changed much slowly without ADWX-1 (Fig. 4B, black).

To better understand the regulatory mechanism of Kv1.3 channels in T cells, we constructed a model T-cell, composed of the Kv1.3 channel, Ca^2+^-release activated calcium (CRAC) channel, intermediate K^+^ (IK or K_Ca_3.1) channel and leak K^+^(TASK) channel [3], to simulate the membrane potentials in a T cell, based on the Eq. (3–5) and the open probability P_o_ of each channels (See the Methods and Materials). For more precise, we calculated the time- and voltage-dependent total conductance of Kv1.3 via the open probability P_o_ of Kv1.3 kinetic model rather than via the formulized term m^3^ h in classic Hodgkin-Huxley model (See Eq. (4)). Essentially, they are the same. According to this T-cell model, our simulations revealed that the total conductance of Kv1.3 was 4.5 nS (N_Kv1.3_ ∼300) for control, 2.0 nS (N_Kv1.3_ ∼133) for 30 nM ADWX-1 and 0.4 nS (N_Kv1.3_ ∼27) for 300 pM ADWX-1 (Fig. 4B). The corresponding membrane potential depolarization property in these situations depended on the number of Kv1.3 channels, indicating that Kv1.3 played a dominative role in maintaining the membrane potential of T cells. In order to further validate the accuracy of the Kv1.3 channel model and T cell model, we used one membrane potential trace recorded from a T cell as a voltage command to stimulate the other T cells, then we obtained a hump-shaped Kv1.3 current, which could be well reproduced by the Kv1.3 kinetic model, by using the same voltage command (Fig. 4C). This showed that the Kv1.3 current started to be activated at about −40 mV and then reached to the maximum at ∼40 mV, and finally decreased to the minimum at more positive voltages due to inactivation. In this simulation, we found that the number of Kv1.3 was about 330, consistent to the previous report [3]. Note that here we subtracted the background current by applying with 1 nM ADWX-1 to get the pure Kv1.3 current.

### The membrane potential of T cells is governed by Kv1.3 channels

As the T cell is a secretory cell [48], it is important to know the intracellular local and global Ca^2+^ signaling. Preferably, it is to know the local Ca^2+^ signaling inside T cells. Since the I-V curve of CRAC channel is substantially linear and the membrane potential is the only driving force to promote the Ca^2+^ influx [26]–[28], we first calculated the membrane potentials in a model T-cell containing different amount of Kv1.3 channels. Fig. 4D illustrated that the changes in membrane potentials of T cells clearly depended on the total conductance (or number) of Kv1.3 channels. Here we chose several typical numbers of Kv1.3 channel in T cell. For example, the total conductance of 22.5 nS implied the number of Kv1.3 was 1500 as a single-channel conductance of Kv1.3 was 15 pS [36]. An activated T cell with 1500 Kv1.3 channels could be considered as an effector memory T_EM_ cell [3].

Afterwards, we further calculated the corresponding local calcium signaling based on Eq. (5). The calculated Ca^2+^ signaling exhibited a Ca^2+^ peak at negative potentials, following by a lower Ca^2+^ plateau at positive potentials (Fig. 4E). The maximal local Ca^2+^ concentration around vesicles is up to over 20 µM, which would be sufficient to induce T-cell exocytosis [45]. Our results revealed that the Ca^2+^ signaling strongly depended on the numbers of Kv1.3 channel. The larger amount of Kv1.3 had the higher Ca^2+^ plateau. In other words, the less Kv1.3, the less [Ca^2+^]_i_ for secretion, suggesting that a T cell lacking of Kv1.3 might lose function. Recently, it was found that the Kv1.3 knockout mice produced lower incidence and severity of T cell-mediated autoimmune encephalomyelitis [49], which was in line with our proposed model that the less Kv1.3 channels would inhibit the calcium entry into T cells and likely further affected the cytokine production. The Kv1.3, IK and CRAC currents in the cases of Figure 4 D–4E were shown in Figure 1. Note that the inactivation property of Kv1.3 channel caused a pseudomorph paradox of the larger conductance conferring the smaller currents, which was actually derived from the smaller open probability due to inactivation of Kv1.3 channel. Nevertheless, K_V_1.3 currents were still much larger than others, indicating that neither CRAC nor IK could significantly affect membrane potentials as K_V_1.3 could. This also suggested that the intracellular Ca^2+^ was dominated by Kv1.3 channels.

The CRAC channel was triggered by IP3-induced depletion of the ER Ca^2+^ store [18]–[20]. In Fig. 4F, however, we found that the 60% lower osmolarity solution induced a global augment of intracellular Ca^2+^ in T cells, consistent to the report by Ross and Cahalan [40]. The low osmolarity solution activated a swelling-activated Cl^−^ (Cl_swell_) channel by swelling T cell, leading depolarization of T cell [3], [50], [51]. The time course of the global Ca^2+^ concentration showed a Ca^2+^ peak at beginning, following by the higher Ca^2+^ plateau, comparable with that as predicted by the model T cell (Fig. 4E). Obviously, the slow effect of the regulatory volume decrease (RVD) could not be the major reason for the Ca^2+^ decay in seconds, because it occurred in minutes. Additionally, it was not clear whether the low osmolarity can directly trigger the opening of the CRAC channel or stretch-activated cation channel with a reversal potential V_rev_≈0 mV. For simulation, there was actually no essential difference between the open of CRAC and stretch-activated cation channels at about −40 mV (<0 mV) in applying with the lower osmolarity solution [3], because the calcium influx derived from either the CRAC or cation channel was governed by Kv1.3 channels.

## Discussion

The effector memory T_EM_ cell, composed of Kv1.3^high^K_Ca_3.1^low^ has been confirmed for several human autoimmune diseases, such as multiple sclerosis (MS), type 1 diabetes mellitus (T1DM) and rheumatoid arthritis (RA) [5]–[7]. The Kv1.3 voltage-gated potassium channel regulates membrane potential and calcium signaling in T_EM_ cells that are key mediators of autoimmune diseases, a thoroughly studying the physiological role of Kv1.3 channel plays in T cell not only helps to understand the working mechanism of T cell, but also provide a crucial clue for treatment of diseases.

In this study, we systematically studied the basic characteristics of Kv1.3 channel and constructed a sequential kinetic model capable to precisely replicate all the kinetics of this channel. Moreover, we examined the Kv1.3 currents in T lymphocyte cell by a specific inhibitor ADWX-1 toxin, consistent to that of expressed in HEK293. To explore the physiological role of Kv1.3 channel, we further built a T model cell, which was composed of four basic channels prominently in T lymphocyte cell. Finally, we demonstrated that the Kv1.3 channels could dominate the membrane potentials of T lymphocyte cell, and that the CRAC channels manipulated the intracellular Ca^2+^ signaling along with changes of membrane potentials.

Using the model T-cell composed of four channels, we have examined the mechanism of Kv1.3 channel regulating T lymphocyte cells. Actually, a couple of channels, i.e., TRPM7, Osmo-activated Cl^−^ (Cl_swell_), and TRPC3/6 were excluded from this T-cell model. The TRPM7 (or a Mg^2+^-inhibitor-Ca^2+^ (MIC) permeable channel) is a stretch- and swelling-activated cation channel, which can be activated by PIP_2_ and inhibited by Mg^2+^ [52], [53]. The Cl_swell_ channel conducts an outwardly rectifying chloride current (or volume-regulated anion current), leading an anion and osmolyte efflux that is activated by cell swelling [54]. In our experiments, TRPM7 current was blocked by intracellular 2.5 mM Mg^2+^. Besides CRAC channel, TRPC3/6 channels provide another Ca^2+^ entry pathway, but it seems that these two channels are little expressed in T cells, the amplitude of TRPC3 inward current is only around 2 pA/pF (or 1–3 pA/cell) at −50 mV [55], which could be neglected in our model, so the model T-cell composed of four basic channels is thus good enough for this work.

During the whole depolarization process of membrane potentials, CRAC contributes much less current than that of Kv1.3, due to the small total conductance of 0.09 nS compared with ∼4.5 nS of Kv1.3. And the membrane potential was much less sensitive to the CRAC channel conductance in simulations, suggesting that Kv1.3 played the key role in dominating membrane potentials in T cells. While CRAC channel was the major Ca^2+^ source in T cells, which provides a continuous calcium influx to elevate the intracellular Ca^2+^ level for T-cell exocytosis. With TASK channels together, Kv1.3 channels also play a role in maintaining the rest potential.

**Figure 5:**
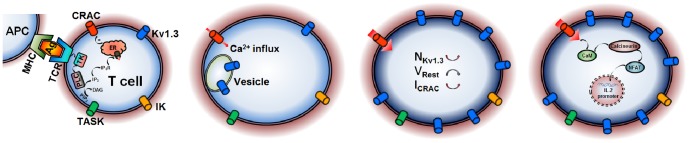
A putative activation mechanism of T cells. Motile polarized T cells naturally use their T-cell receptor (TCR) to come into contact with antigen(Ag)-presenting cell (APC) via peptide–major histocompatibility complex (MHC); T cells tagged activate the smaller amount of Ca^2+^ influx via CRAC channel, probably evoked by IP_3_-induced depletion of Ca^2+^ stores, to slightly increase the expression of Kv1.3; more Ca^2+^ influx via CRAC channel enters into T cell due to membrane repolarization by augment of Kv1.3; The higher Ca^2+^ in T cell triggers the IL-2 promoter production program.

In this study, we identified the physiological role of Kv1.3 channels, which further provided us an opportunity to better understand the activation mechanism of T cells. How can a CRAC channel in a T cell be activated following antigen stimulation? In Fig. 5, a cartoon shows that an activated T cell increases the surface expression of Kv1.3, to negative-shift the membrane potential to increase the intracellular Ca^2+^ via CRAC channels, and finally to start the IL-2 (interleukin 2) promoter production program [3]. This process is more like a positive feedback [3], [18], possibly terminated by endocytosis due to internationalization of Kv1.3 channels. This will decrease the number of Kv1.3 on plasma membrane to lead T cell gradually return to its resting state again.

In this study, we provided a precise model of Kv1.3 and a novel non-excitable model cell for quantitatively investigating the regulatory mechanism of T cell, which laid a solid basis for further studying other channel such as IK, TASK roles in T cell and the events of membrane potentials, calcium signaling and secretion in other non-excitable cells such as T_CM_, B and sperm cells.
